# Pregnancy outcomes at Mizan-Tepi University Teaching Hospital: A Comparison to the Ethiopian Demographic and Health Surveys

**DOI:** 10.26502/ogr056

**Published:** 2021-04-14

**Authors:** Margo Shawn Harrison, Margaret Muldrow, Ephrem Kirub, Tewodros Liyew, Biruk Teshome, Andrea Jimenez-Zambrano, Teklemariam Yarinbab

**Affiliations:** 1 University of Colorado School of Medicine, Aurora, Colorado, USA; 2 Village Health Partnership, Denver, Colorado, USA; 3 Mizan-Tepi University Teaching Hospital, College of Health Sciences, Mizan-Aman, Ethiopia

**Keywords:** Pregnancy Outcomes, Ethiopia, Sub-Saharan Africa

## Abstract

**Background:**

To compare outcomes at Mizan-Tepi University Teaching Hospital to national and regional data and to plan quality improvement and research studies based on the results.

**Methods:**

This study was a prospective hospital-based cross-sectional analysis of a convenience sample of 1, 000 women who delivered at Mizan-Tepi University Teaching Hospital.

**Results:**

Our convenience sample was young (median age 24 years) with a primarily school level or less of education (68.6%). Only about 5% of women had a history of prior cesarean birth, 2.1% reported they were human immunodeficiency virus seropositive, and the median number of prenatal visits was four. Women were commonly admitted in spontaneous labor (84.5%), transferred from another facility (49.2%; 96.8% of which were referred from a health center), and had their fetal heart rate auscultated on admission (94.7%). Only 5.2% of women did not deliver within twenty-four hours and the cesarean birth prevalence was 23.4%. Many women were delivered by midwives (73.2%; all unassisted vaginal births), 89.2% were term deliveries, and 92.5% of neonatal birthweights were 2500 grams or heavier. Less than five percent of women delivered stillbirths (4.3%) and 5.7% of livebirths experienced neonatal death by the day of discharge. There were no maternal deaths in the cohort.

**Conclusion:**

The prevalence of stillbirth and neonatal death were the most notable findings, while there was no maternal death in the cohort.

## 1. Background

Ethiopia is an East African, landlocked, multi-ethnic country with the twelfth largest population in the world [1]. Ethiopia experiences a high health burden and is home to one of the world’s largest populations of people living in povery [2]. A recent Mini Demographic and Health Survey (MDHS) was conducted between March and June 2019 by the Ethiopian Public Health Institute in partnership with the Federal Ministry of Health and the Central Statistical Agency [3]. The intent of the survey was to provide current health and demographic data on maternal and child health to identify target areas for strategic health improvement initiatives [3]. Given this recent publication of high quality data, the intent of our study was to determine how pregnancy outcomes at Mizan-Tepi University Teaching Hospital compare nationally and regionally to both the MDHS and the full Ethiopian Demographic and Health Survey performed in 2016 (2016 DHS) [4].

This manuscript presents the results of a cross-sectional inpatient survey of admission, delivery, and discharge data on 1, 000 women giving birth at a teaching and referral facility in rural, Southwest Ethiopia. The intent of this survey is similar to the MDHS in that we aim to identify target pregnancy outcomes for prospective quality improvement and research interventions to focus our initiatives at the hospital, and in the surrounding catchment area, in order to improve maternal and child health.

## 2. Methods

### 2.1 Study setting

The survey was conducted at Mizan Tepi University Teaching Hospital (MTUTH), which is located in Mizan-Aman in the Southern Nations, Nationalities, and People’s Region (SNNPR). The hospital serves a catchment population of 2.75 million people. The labor and delivery unit at the hospital is the only obstetric unit capable of providing cesarean birth within 100 kilometers. In 2018, about 2880 women gave birth at MTUTH.

### 2.2 Study design

This study was a prospective, hospital-based cross-sectional study of a convenience sample of 1, 000 women. The objective of the survey was to collect sociodemographic, obstetric, and outcomes data in order to establish baseline rates of pregnancy outcomes and to identify target areas for quality improvement and research interventions.

### 2.3 Study population

The source population for this study was all pregnancy women with visited MTUTH for service during the study period, while the study population was a convenience sample of pregnant women who delivered on labor and delivery at MTUTH between May 6 and October 21, 2019. Only mothers who delivered after 28 completed weeks of pregnancy were included.

### 2.4 Data collection procedures

This quality improvement dataset was designed by all authors and based on prior World Health Organization Global Surveys and the Eunice Kennedy Shriver Global Network for Women’s and Children’s Health Research forms and procedures [5–7]. De-identified data was collected by highly trained physician data collectors with the intent of planning future quality improvement and research interventions. In order to provide complete data on all births that took place in the study timeframe, data collectors visited the labor and delivery ward four times per day (8:00AM, 1:30PM, 4:30PM, 8:00PM). A combination of structured interview and chart review was used to collect information upon admission, delivery, and discharge. Data was collected on paper forms and the data collectors reviewed each other’s forms for completeness prior to data entry into REDCap for transmission and secure storage on a password protected server at the University of Colorado, Aurora, Colorado, USA [8].

### 2.5 Data management

Until data was entered into the electronic data capture software, paper forms were stored in a storage cabinet, inside a locked room to which only one of the data collectors had access.

### 2.6 Data analysis

Data from the paper forms were entered into REDCap 9.1.9 STATA software version 15.2 (StataCorp LP, College Station, TX, USA) was used for analysis. Frequency and percentage tables were utilized to depict distribution of the variables.

### 2.7 Ethical approval

This quality improvement survey was given an exempt from human subjects’ research approval (COMIRB # 18–2738) by the University of Colorado. Despite the quality improvement nature of the work and the fact that only de-identified data was collected, oral consent was obtained from each woman before any of her data was recorded. Approval was also granted by Mizan-Tepi University Teaching Hospital.

## 3. Results

### 3.1 Sociodemographic characteristics

Less than 1% of data was missing on the sociodemographic characteristics of our convenience sample, which can be found in Table 1. The women giving birth at MTUTH were young adults (median age 24 years, interquartile range (IQR) 20 – 28 years; mean age 24.6 years, standard deviation (SD) 4.7 years). The majority of women had a primary school level of education or less (68.6%; 23.3% were illiterate), but 17.3% had achieved higher education. The majority of women were of Protestant religion (54.9%) followed by Orthodox Christian (33.6%). Most women were either married or cohabitating (96.4%), and less than 1% of women reported using tobacco or alcohol.

### 3.2 Regional representation

Almost half of the women in our cohort (45.4%) hailed from the community surrounding the hospital, Mizan-Aman. The next largest contingent was from the South Bench region (15.6%), with all other areas contributing less than 10% (Table 1). Gidi Bench contributed the smallest number of women to the analysis (n = 3, 0.3%).

### 3.3 Obstetric and medical characteristics

Among our cohort, 42.8% of women were nulliparous and 5.8% were grand multiparous (parity of at least five). The median interpregnancy interval (time since last pregnancy outcome) was 56 months and the mean interval was 60.7 months; 91.7% of the sample achieved the World Health Organization (WHO) recommended pregnancy spacing of 24 months between livebirth and subsequent conception [9]. Around half of women reported an estimated due date determined by ultrasound (47.2%) compared to 45.7% of women dated by clinical exam, and 6.6% dated by last known menstrual period.

A history of prior cesarean birth was reported by 4.4% of the cohort, and 0.5% of the population had had two or more prior cesarean births. Two women reported a history of myomectomy (uterine surgery to remove leiomyomata) and no women reported a history of uterine rupture. Twenty-one women (2.1%) were reportedly human immunodeficiency virus seropositive, less than 1% of women were anemic by self-report, and only two women reported a history of gestational or pregestational diabetes. The cohort was seen for a median of 4 prenatal visits with 5.5% of the population achieving the WHO recommended eight antenatal contacts [10]. Results are shown in Table 2.

### 3.4 Labor and delivery characteristics

Eight-hundred and forty-five women were reportedly admitted to MTUTH in spontaneous labor with 12.3% of the cohort requiring augmentation or induction. Almost half of the population (49.2%) was referred to MTUTH from another facility, which was a health center in 96.8% of cases. Two-thirds of women admitted in labor were in the latent stage (cervical exam less than 6 centimeters [cm] in accordance with newer definitions), with a median cervical dilation at admission of 3 cm (IQR 2 – 7 cm); however, MTUTH the active stage is defined as 4cm according to traditional definitions [11, 12]. The fetal heart rate (FHR) was auscultated on admission in 94.7% of cases; in 4.1% it was not heard due to fetal demise, and in only 2 cases was the FHR on admission not documented. Labor duration (in the hospital) was complete in less than 24 hours in 94.7% of the population, with only 5.2% of the sample progressing past a full day.

In the case of 61.9% of women, the partograph was reportedly “completely” utilized and was noted as “not applicable” in 27.2% of records. The median number of vaginal exams performed over the course of labor was 3 (IQR 2 – 3 exams). Regarding mode of delivery, 71.6% of women experienced an unassisted vaginal birth, 1.4% were delivered vaginally with forceps assistance, 2.9% required vacuum assistance, and cesarean birth was performed in 23.4% of births. 9.8% of cesareans were “electively” performed and 95.8% of women undergoing cesarean received appropriately timed antibiotic prophylaxis. Anesthesia was not given in 67.4% of women (vaginal births), while 22.0% were anesthetized with a spinal and 1.3% of women received general anesthesia (cesarean births). The delivering provider was a midwife in 73.2% of cases of spontaneous vaginal birth with the majority of the remainder delivered by integrated emergency and surgical officers (IESO, 24.3%) [13]. Most women had term births (89.2%), 92.5% delivered infants weighing greater than or equal to 2500 grams, 53.1% of the infants were male (5.4% missing data), and 4.9% of pregnancies were multiple gestation. The median five-minute Apgar score was 9 (IQR 8, 9). Results can be found in Tables 3 and 4.

### 3.5 Pregnancy outcomes

Maternal Antepartum Complications: Antepartum complications occurred in a minority of the population with 2.2% of women experiencing antepartum hemorrhage, 0.3% requiring an antepartum blood transfusion, 0.6% diagnosed with chorioamnionitis, 3.3% treated with antepartum antibiotics, 4.7% diagnosed with an antepartum hypertensive disorder of pregnancy, 1.1% requiring antepartum hypertensive treatment, and 1.8% treated with antepartum magnesium sulfate (for maternal indications). Antepartum, preterm steroid treatment was administered during the antepartum course of 0.6% of the sample. Results are shown in Table 5.

### 3.6 Postpartum maternal outcomes (Table 6)

Postpartum hemorrhage occurred in 0.4% of births, uterotonics were administered in 0.2%, there were reportedly no postpartum dilation and curettage procedures performed, and postpartum blood transfusion was given to 1.5% of women. Regarding infection, 2 women had a wound infection, none had a wound dehiscence, 2 women were treated for urinary tract infection, none were diagnosed with pneumonia, and 8 women were treated for postpartum endometritis (0.8%). However, postpartum antibiotic administration was delivered to 9.0% (90 women) of the sample. 24 women were treated with an antihypertensive and 26 with an anticonvulsant. One woman was referred to the intensive care unit, but was not admitted, all women were alive 24 hours after birth and at discharge, and the median hospital stay was 1 day (IQR 1, 3).

### 3.7 Postpartum neonatal outcomes (Table 7)

Around 5% of the neonatal outcome variables were missing data. Of women for whom data was available (n = 947), 4.3% delivered stillbirths; 30 were fresh stillbirths and 13 were macerated. Bag and mask resuscitation was used in 1.2% of cases, oxygen by way of nasal cannula was given to 2.0% of infants, and continuous positive airway pressure (CPAP) was administered to 0.9% of the sample. Intravenous fluids were given to 3.1% of babies, antibiotics were administered to 3.4%, blood transfusion was given in 0.4% of cases, and 5.7% of infants died by the day of discharge (median hospitalization 1 day, IQR 1 – 3 days).

## 4. Discussion

In summary, in our cross-sectional analysis of a convenience sample of 1, 000 women who gave birth at MTUTH, half of women were transferred to the facility, most women delivered in less than 24 hours after admission, the majority of births were term with a healthy birthweight infant, and rates of hypertensive disease and hemorrhage were not high. The prevalence of stillbirth and neonatal death were the most notable findings, and there was no maternal death in the sample. We wish to compare these findings to the national and regional health outcomes data provided by the MDHS and the 2016 DHS, and published literature—primarily the African cohort in the World Health Organization Global Survey on Maternal and Perinatal Health (WHOGS) and the African subgroup in the World Health Organization Multicountry Survey on Maternal and Newborn Health (WHOMCS) [5, 6]. Regarding sociodemographics, compared to the MDHS population (which was 19.2% from SNNPR), our population was less Muslim, more Protestant, and less single (66.0% of the national population was not single, compared to 96.4% of our population) [3]. Just under half the women in our cohort live in an urban area (45.4%) compared to 32.3% in the MDHS [3]. Our population had a higher proportion of women with higher education and a lower proportion of women with no education compared to the national sample [3]. Therefore, we believe our cohort was married and more educated than the average Ethiopian in the region.

The obstetric characteristics of our cohort varied compared to the literature. In our cohort, the median parity was 1, which was lower than the median parity of 2 for all African women in the WHOGS cohort [5]. Concerning birth spacing, the median birth interval in the 2016 DHS was 34.5 months compared to a longer median of 56 months in our study cohort [4]. Data on method of gestational age determination (last menstrual period versus clinical exam versus ultrasound) in other Ethiopian cohorts was not found, but we did find another inpatient Eastern Ethiopian cohort where the number of elective/planned cesarean births (used as a proxy for history of cesarean birth) was 7.4%, compared to 4.9% of women in our population and 4.2% in WHOGS with a history of cesarean birth [5, 14]. Finally, compared to the MDHS, where 34.1% of women of reproductive age received four or more prenatal visits during the course of any pregnancy leading to a livebirth in the last five years (69.4% with a skilled provider), 68.3% of our population reported four or more visits with an antenatal care nurse or midwife [3]. Our results suggest that women in our cohort have low parity and are achieving good interpregnancy intervals. However, care could be improved by increasing the number of women whose pregnancies are dated by ultrasound and by increasing antenatal visits to the WHO-recommended eight contacts [10]. The other notable finding is the low rate of history of cesarean birth; while a 23.4% rate of cesarean in the hospital seems in the realm of appropriate given that most women are delivering within 24 hours (though we cannot determine this without further information), the less than 5% rate of history of cesarean birth, though consistent with other data sources, suggests that access to cesarean birth in the region may be low [5, 14].

We also found variation in the medical characteristics of our sample compared to the literature. The Center for Disease Control and Prevention in the United States of America estimates that as of fiscal year 2018, 1% of the Ethiopian population was living with human immunodeficiency virus (HIV); among our study cohort of pregnant women the prevalence was higher at 2.1%, but similar to the prevalence to African in WHOGS (1.9%) [15]. With respect to the diagnosis of anemia, 24% of women were anemic in the 2016 DHS, where only 0.4% of women self-reported being anemic, although severe anemia reported in WHOGS was 0.7% in the African subgroup [4, 5]. The prevalence of gestational and pregestational diabetes in sub-Saharan Africa has previously been reported as between 0 – 13.9% of the pregnant population; the 0.2% prevalence in our population falls within that range and is similar to the 0.4% prevalence reported by WHOGS [5]. If better HIV, hemoglobin, and glucose testing was available, we might see increased prevalence of disease, which would offer opportunities for improved management during labor and delivery, and potentially improved outcomes as a result. Nearly 85% of women that presented to MTUTH were admitted in labor, which is similar to WHOGS, which reported 92.2% of African women in the sample were admitted in labor [5]. We did note that two-thirds of women were in latent labor when admitted, but the majority still delivered within twenty-four hours, suggesting that most labors progressed appropriately, though less than two-thirds of partographs were completed. The partograph has been shown, in an updated systematic review, to help with intrapartum decision-making and to reduce stillbirth [16]. Stillbirth is high in this cohort, so improved partograph completion, adherence, and actual usage during labor might be a target area to improve neonatal outcomes. Another notable quality metric was that only 2 women did not have their fetal heart rate auscultated on admission; it has been shown that performance of auscultation on admission and notation of a fetal heart rate is associated with a reduced perinatal mortality rate, so the hospital is performing well on this measure [16, 17].

Regarding mode of birth, while forceps- and vacuum-assisted vaginal births accounted for less than 5% of births, this is consistent with findings from other low- and middle-income countries, including sub-Saharan African sites [18, 19]. Interpretation of the cesarean birth rate of 23.4% requires further evaluation. While higher than the traditionally recommended 10 – 15% cesarean birth rate by WHO, the rate is high enough to prevent undue morbidity and mortality and is close to the 19% recommended by some authors as the ideal cesarean birth rate [20–22]. Compared to an Eastern Ethiopian inpatient cohort, the cesarean birth rate was similar to their rate of 25.7%, 92.6% of which were unplanned (compared to our 90.2%) [14]. They reported an 11.3% preterm birth rate compared to our 10.4 (11.5% in WHOGS), and they experienced a 16.1% prevalence of low birthweight among their infants compared to our 7.5% (9.6% in WHOGS) [14]. Our multiple birth rate was around 4.9/1000 while previously published numbers from Ethiopia are slightly higher at 11.7/1000, with the overall rate in African sites in WHOGS being 3.5% [5, 23]. The appropriateness of the cesarean birth rate requires further study, but if it is determined that the rate is too high, improving use of forceps and vacuum usage might be one method to reduce rates. Additionally, while there are currently minimal interventions to reduce preterm birth, when the results of a trial assessing the impact of low-dose Aspirin® are published, if positive, a public health program to increase early utilization of the medicine may have an impact on preterm birth rates at MTUTH [24].

Regarding antepartum maternal complications, our rate of antepartum hemorrhage was 2.2% compared to 4.3% in another Ethiopian cohort [25]. Women requiring an antepartum blood transfusion was 0.3% (3.7% in WHOGS) in our sample, and our antepartum antibiotic administration was 3.3% compared to 17.4% in WHOGS, though there are likely some definitional differences in these comparative results. Our antepartum pre-eclampsia/eclampsia/chronic hypertension prevalence was 4.7% compared to a “pregnancy induced hypertension” prevalence of 2.6% and a 2.5% prevalence of pre-eclampsia/eclampsia/chronic hypertension in WHOGS (the latter was 2.7% in WHOMCS) [5, 26]. Corticosteroids were administered in 0.6% of our women antenatally compared to 52% in WHOMCS, though this may reflect the fact that trials questioning the safety of antenatal corticosteroids in low-resources settings have been published since that time [27, 28]. Further studies evaluating use of antenatal corticosteroids in low-resource settings will further inform these findings, and as WHO recommendations evolve, we will consider increasing use of this intervention at MTUTH [29].

One of our most interesting findings was the low rate of postpartum hemorrhage in our cohort, which was 0.4% in our sample (where prophylactic uterotonics are administered) compared to 16.6% in other Ethiopian settings; the rate was 1.2% in WHOMCS when prophylactic uterotonics were used [30, 31]. Similarly, from a clinical perspective, it is surprising that no dilation and curettage procedures were necessary in our patient cohort, as this is not an uncommon procedure in the setting of hemorrhage or retained products of conception; other authors in sub-Saharan Africa have reported a 1.9% prevalence [32]. Blood transfusion was administered in 1.5% of our cohort compared to 20.6% of the African subgroup in WHOGS experiencing hemorrhage, and use of postpartum antibiotics was 9.0% in our sample compared to 36.9% of vaginal births and 21.2% of cesarean birth in WHOGS [5]. Infection timing in our cohort versus others, as with other variables, is difficult to determine given the varying definitions and multiple time points at which data was collected in different settings and the short median hospital stay in our women; urinary tract infection was clear, however, and was 0.2% prevalent in our women compared to 3.6% in WHOGS.^5^ Our other outcomes, including referral and admission to the intensive care uni, and maternal death essentially did not occur (compared to a 0.3% maternal death prevalence in WHOGS) [5]. These findings suggest that we may want to further evaluate the prevalence and management of postpartum hemorrhage at MTUTH; we may be underdiagnosing the complication, or, if the 0.4% is true, we need to understand why so we can share the findings with other, similar settings. Regarding neonatal outcomes, our stillbirth rate was 43 of 947 births (45.4 per 1000, 4.5%) compared to a prevalence of 3.7% in the African cohort in WHOGS.^5^ The 2019 MDHS reports 30 neonatal deaths per 1000 live births [3]. In our cohort, where infants were only followed until discharge and not through 28 days of life, we had 14 of 903 liveborn neonates succumb during their hospitalization (15.5/1000). Respiratory support was used in 2% or less of our infants, fluids and antibiotics were given in 3% of cases, and blood transfusion in 0.4%. We believe that neonatal outcomes should be a focus at MTUTH moving forward; reducing the stillbirth rate through effective intrapartum interventions (like the previously discussed partograph) and better understanding risk factors contributing to neonatal demise so evidence-based interventions can be put in place to reduce this adverse outcome.

## 5. Conclusion

This descriptive analysis was performed to generate hypotheses about how we might improve maternal and child health at MTUTH and in the surrounding regions. It is limited by missing data on socioeconomic status, and the fact that we did not collect an extensive set of variables, which may have provided for more rich secondary analyses of particular outcomes of interest. For example, we had a low prevalence of infections, which is likely a reflection of the short median hospital stay rather than an overall low prevalence of infectious complications. The strengths of this analysis are the relatively large sample size, the very small amount of missing data, the high-quality of data collection, and the representativeness of an underrepresented region of Ethiopia. On review of our data compared to the literature, areas of interest for further evaluation include better understanding the following outcomes: increasing antenatal attendance closer to eight antenatal contacts, improving pregnancy dating, improving high-quality partograph use, determining the appropriateness of the cesarean birth rate, evaluating the validity of the data regarding postpartum hemorrhage, and improving fetal and neonatal outcomes in terms of reducing the stillbirth and neonatal death rate.

## Figures and Tables

**Table 1:** Sociodemographic Characteristics of Convenience Sample of Women, Mizan-Tepi University Teaching Hospital, Ethiopia, 2019.

Characteristic	N (%) N = 1000
Age in years [Median (IQR); Mean (SD)]	24 [20, 28], 24.6 ± 4.7
Missing	1 (0.1%)
**Education**	
Unable to read & write	233 (23.3%)
Read & write only	54 (5.4%)
Primary school	399 (39.9%)
Secondary school	140 (14.0%)
Higher education	173 (17.3%)
Missing	1 (0.1%)
**Religion**	
Muslim	111 (11.1%)
Orthodox Christian	336 (33.6%)
Catholic Christian	0 (0.0%)
Protestant	549 (54.9%)
Jehovah’s Witness	2 (0.2%)
Missing	2 (0.2%)
**Relationship Status**	
Single	27 (2.7%)
Not single	964 (96.4%)
Missing	9 (0.9%)
**Residence**	
Bero	74 (7.4%)
Gidi Bench	3 (0.3%)
Gura Ferda	62 (6.2%)
Menet Goldia	23 (2.3%)
Menet Shasha	9 (0.9%)
Mizan-Aman	454 (45.4%)
North Bench	37 (3.7%)
Shebench	78 (7.8%)
Sheko	98 (9.8%)
South Bench	156 (15.6%)
Surma	6 (0.6%)
Missing	0 (0.0%)
**Mother uses Tobacco**	
Yes	1 (0.1%)
No	994 (99.4%)
Missing	5 (0.5%)
**Mother uses Alcohol**	
Yes	5 (0.5%)
No	990 (99.0%)
Missing	5 (0.5%)

**Table 2:** Obstetric and Medical History of Convenience Sample of Women, Mizan-Tepi University Teaching Hospital, Ethiopia, 2019.

Characteristic	N (%) N = 1000
**Parity**	
0	428 (42.8%)
1	263 (26.3%)
2	144 (14.4%)
3	68 (6.8%)
4	37 (3.7%)
5+	58 (5.8%)
Missing	2 (0.2%)
**Months Since Last Delivery (parity 1+ n = 572)**	
< 24 months	46 (8.1%)
24+ months	525 (91.7%)
Missing	1 (0.2%)
Median (IQR); Mean (SD)	56 [36, 84]; 60.7 ± 34.6
**Gestational Age Determination**	
Clinical Exam (Fundal Height)	457 (45.7%)
Last Menstrual Period	66 (6.6%)
Ultrasound	472 (47.2%)
Missing	5 (0.5%)
**History of Cesarean Birth**	
0	949 (94.9%)
1	44 (4.4%)
2+	5 (0.5%)
Missing	2 (0.2%)
**History of Myomectomy**	
Yes	2 (0.2%)
No	996 (99.6%)
Missing	2 (0.2%)
**History of Ruptured Uterus**	
Yes	0 (0.0%)
No	995 (99.5%)
Missing	5 (0.5%)
**HIV+**	
Yes	21 (2.1%)
No	970 (97.0%)
Missing	9 (0.9%)
**Anemic**	
Yes	4 (0.4%)
No	995 (99.5%)
Missing	5 (0.5%)
**Pregestational/Gestational Diabetes**	
Yes	2 (0.2%)
No	996 (99.6%)
Missing	2 (0.2%)
**Number of Prenatal Visits**	
0	21 (2.1%)
< 8	919 (91.9%)
8+	55 (5.5%)
Missing	5 (0.5%)
Median (IQR); Mean (SD)	4 [3, 5]; 4.2 ± 2.1

**Table 3:** Labor Characteristics of Convenience Sample of Women, Mizan-Tepi University Teaching Hospital, Ethiopia, 2019.

Characteristic	N (%), N = 1000
**Onset of Labor**	
Spontaneous	845 (84.5%)
Augmented/Induced	123 (12.3%)
Not Applicable	28 (2.8%)
Missing	4 (0.4%)
**Transferred During Labor**	
Yes	492 (49.2%)
No	506 (50.6%)
Missing	2 (0.2%)
**If Transferred, Transferring Facility (Transferred n = 492)**	
Health Center	476 (96.8%)
Private Clinic	7 (1.4%)
Primary Hospital	3 (0.6%)
Missing	6 (1.2%)
**Cervical Exam on Admission**	
<6 cm (latent labor)	660 (66.0%)
6+ cm (active labor)	308 (30.8%)
Missing or “Not done” or “Not Applicable”	32 (3.2%)
Median (IQR); Mean (SD)	3 [2, 7]; 4.5 ± 3.1
**Fetal Heart Rate Auscultated at Admission**	
Yes	947 (94.7%)
No (Fetal Demise)	41 (4.1%)
Not Auscultated	2 (0.2%)
Missing	10 (1.0%)
**Duration of Labor**	
Not Applicable	33 (3.3%)
< 12 hours	512 (51.2%)
12 – 24 hours	402 (40.2%)
24+ hours	52 (5.2%)
Missing	1 (0.1%)
**Partograph Used**	
Yes, Complete	619 (61.9%)
Yes, Incomplete	10 (1.0%)
No	94 (9.4%)
Not Applicable	272 (27.2%)
Missing	5 (0.5%)
Number of Vaginal Exams [Median (IQR); Mean (SD)]	3 [2, 3]; 2.7 ± 1.4
Missing	8 (0.8%)

**Table 4:** Delivery Characteristics of Convenience Sample of Women and Infants.

Characteristic	N (%), N = 1000
**Mode of Birth**	
Unassisted Vaginal Birth	716 (71.6%)
Forceps-Assisted Vaginal Birth	14 (1.4%)
Vacuum-Assisted Vaginal Birth	29 (2.9%)
Cesarean Birth	234 (23.4%)
Missing	7 (0.7%)
**Urgency of Cesarean Birth (cesarean birth n = 234)**	
Elective	23 (9.8%)
Emergency	211 (90.2%)
Missing	(0.0%)
**Antibiotics Given Within 60 Minutes of Cesarean Incision (cesarean birth n = 234)**	
Yes	224 (95.8%)
Missing	5 (2.1%)
**Anesthesia for Birth**	
No	674 (67.4%)
Local for vaginal repair	88 (8.8%)
Spinal	220 (22.0%)
Epidural	0 (0.0%)
Combined spinal/epidural	1 (0.1%)
General Anesthesia	13 (1.3%)
Missing	4 (0.4%)
**Delivery Provider**	
Midwife	732 (73.2%)
General Practitioner	10 (1.0%)
Integrated Emergency and Surgical Officer	243 (24.3%)
Ob/Gyn Resident	5 (0.5%)
Ob/Gyn Attending	5 (0.5%)
Missing	5 (0.5%)
**Gestational Age at Delivery**	
Preterm	104 (10.4%)
Term	892 (89.2)
Missing	4 (0.4%)
Median (IQR); Mean (SD) in weeks	39 [38, 40]; 38.7 ± 2.3
**Birthweight (grams)**	
<2500	71 (7.5%)
≥ 2500	875 (92.5)
Missing	54 (5.4%)
Median (IQR)	3100 [2900, 3500]
Mean (SD)	3120.4 ± 548.4
**Neonatal Sex**	
Male	531 (53.1%)
Female	415 (41.5%)
Missing	54 (5.4%)
**Multiple Gestation**	
Yes	49 (4.9%)
Missing	3 (0.3%)
Five-Minute Apgar Score Median (IQR)	9 [8, 9]
Missing	52 (5.2%)

**Table 5:** Antepartum Complications of Labor and Delivery of a Convenience Sample of Births, Mizan-Tepi University Teaching Hospital, Ethiopia, 2019.

Characteristic	N (%), N = 1000
**ANTEPARTUM**	
**Antepartum Hemorrhage**	
Yes	22 (2.2%)
No	976 (97.6%)
Missing	2 (0.2%)
**Antepartum Blood Transfusion**	
Yes	3 (0.3%)
No	994 (9.4%)
Missing	3 (0.3%)
**Chorioamnionitis**	
Yes	6 (0.6%)
No	993 (99.3%)
Missing	1 (0.1%)
**Antepartum Antibiotic Therapy**	
Yes	33 (3.3%)
No	964 (96.4%)
Missing	3 (0.3%)
**Pre-eclampsia/Eclampsia/Chronic Hypertension**	
Yes	47 (4.7%)
No	951 (95.1%)
Missing	2 (0.2%)
**Antepartum Hypertensive Therapy**	
Yes	11 (1.1%)
No	985 (98.5%)
Missing	4 (0.4%)
**Antepartum Magnesium Sulfate Therapy**	
Yes	18 (1.8%)
No	978 (97.8%)
Missing	4 (0.4%)
**Antepartum Steroid Therapy**	
Yes	6 (0.6%)
No	990 (99.0%)
Missing	4 (0.4%)

**Table 6:** Postpartum Maternal Complications of Labor and Delivery of a Convenience Sample of Births, Mizan-Tepi University Teaching Hospital, Ethiopia, 2019.

POSTPARTUM	
**Maternal Outcomes**	
**Postpartum Hemorrhage**	
Yes	4 (0.4%)
No	993 (99.3%)
Missing	3 (0.3%)
**Uterotonic Administration**	
Yes	2 (0.2%)
No	992 (99.2%)
Missing	6 (0.6%)
**Dilation & Curettage**	
Yes	0 (0.0%)
No	988 (98.8%)
Missing	12 (1.2%)
**Postpartum Blood Transfusion**	
Yes	15 (1.5%)
No	980 (98.0%)
Missing	5 (0.5%)
**Infection**	
Wound Infection	2 (0.2%), 0.3% missing
Wound Dehiscence	0 (0.0%), 0.4% missing
Urinary Tract Infection	2 (0.2%), 0.3% missing
Pneumonia	0 (0.0%), 0.3% missing
Endometritis	8 (0.8%), 0.3% missing
**Postpartum Antibiotics**	
Yes	90 (9.0%)
No	902 (90.2%)
Missing	8 (0.8%)
**Postpartum Hypertensive Treatment**	
Antihypertensive	24 (2.4%), 0.5% missing
Anticonvulsant	26 (2.6%), 0.5% missing
**Referral to Intensive Care Unit**	
Yes	1 (0.1%)
No	992 (99.2%)
Missing	7 (0.7%)
**Admission to Intensive Care Unit**	
Yes	0 (0.0%)
No	995 (99.5%)
Missing	5 (0.5%)
**Maternal Status at 24 hours Postpartum**	
Dead	0 (0.0%)
Alive	995 (99.5%)
Missing	5 (0.5%)
**Maternal Status at Discharge**	
Dead	0 (0.0%)
Alive	994 (99.4%)
Missing	6 (0.6%)
Maternal Length of Hospitalization [Median (IQR); Mean (SD)]	1 [1, 3], 1.9 ± 1.9
Missing	7 (0.7%)

**Table 7:** Postpartum Neonatal Complications of Labor and Delivery of a Convenience Sample of Births, Mizan-Tepi University Teaching Hospital, Ethiopia, 2019.

POSTPARTUM	
**Neonatal Outcomes**	
**Stillbirth**	
Yes, Fresh	30 (3.0%)
Yes, Macerated	13 (1.3%)
No	904 (90.4%)
Missing	53 (5.3%)
**Bag & Mask Resuscitation**	
Yes	12 (1.2%)
No	981 (98.1%)
Missing	7 (0.7%)
**Intranasal Oxygen**	
Yes	20 (2.0%)
No	974 (97.4%)
Missing	6 (0.6%)
**Continuous Positive Airway Pressure (CPAP)**	
Yes	9 (0.9%)
No	984 (98.4%)
Missing	7 (0.7%)
**Intravenous Fluid Administration**	
Yes	31 (3.1%)
No	963 (96.3%)
Missing	6 (0.6%)
**Antibiotics**	
Yes	34 (3.4%)
No	956 (95.6%)
Missing	10 (1.0%)
**Blood Transfusion**	
Yes	4 (0.4%)
No	986 (98.6%)
Missing	10 (1.0%)
**Neonate Status on Day of Discharge**	
Dead*	57 (5.7%)
Alive	935 (93.5%)
Missing	8 (0.8%)
Neonatal Length of Hospitalization [Median (IQR); Mean (SD)]	1 [1, 3], 1.9 ± 2.1
Missing	25 (2.5%)

* Total number dead, including stillbirths

## What is Known

- Pregnancy outcomes at a regional, general level in the Southern Nations Nationalities People’s Region of Ethiopia

## What this Study Adds

- Significant detail on pregnancy outcomes at the referral facility in Bench Maji Zone
- Comparison of those results to the national datasets
- More granular detail on sociodemographic, antepartum, intrapartum, and postpartum characteristics than the national datasets
